# Supplementary figures and images for: Bioinformatic analysis identified novel candidate genes with the potentials for diagnostic blood testing of primary biliary cholangitis
Source: PLoS One. 2023 Oct 16;18(10):e0292998. doi: 10.1371/journal.pone.0292998 (PMC10578581; doi:10.1371/journal.pone.0292998)

# S1 Fig

Before outlier replacement

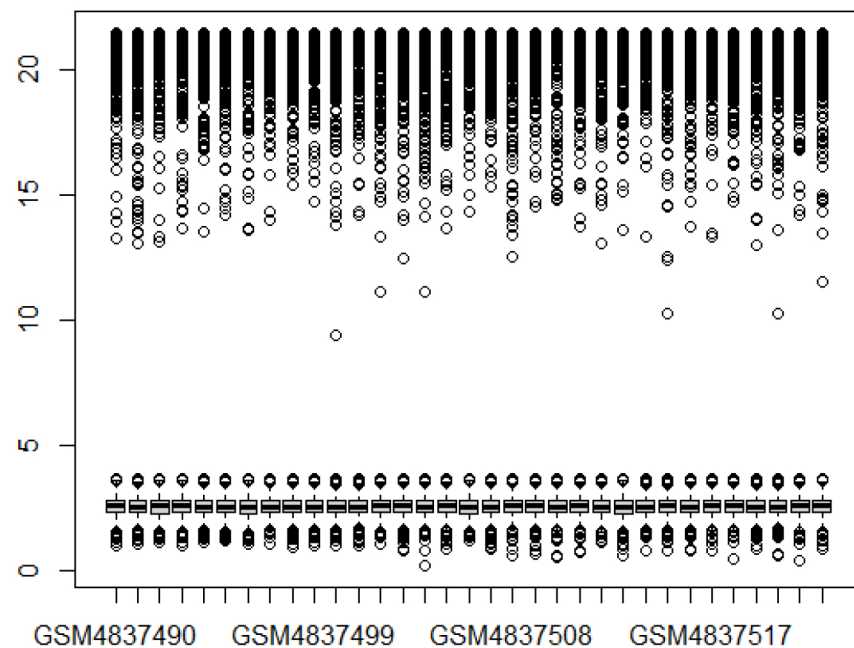

After outlier replacement

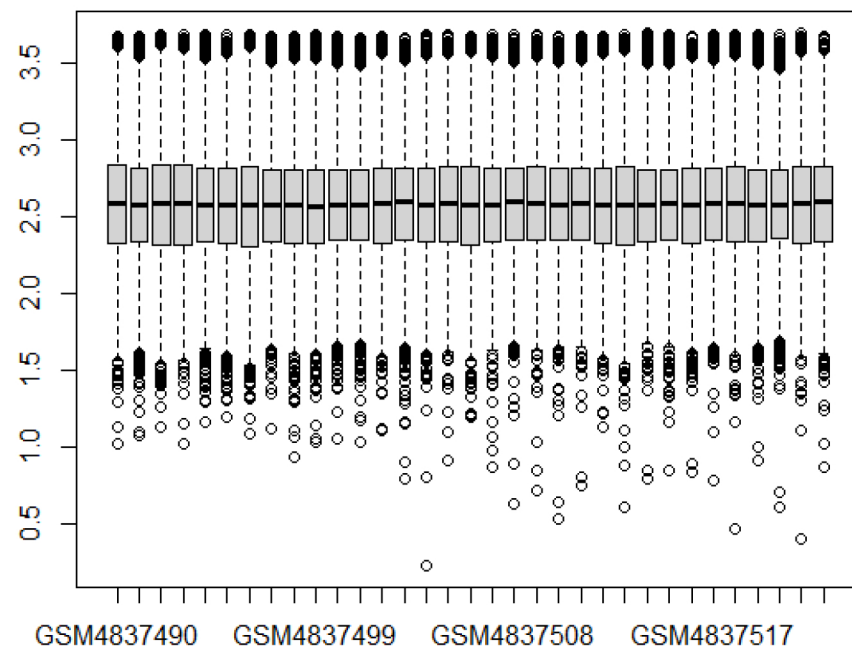

Supplement: S1 Fig — (PDF) [file pone.0292998.s001.pdf]

S2 Fig

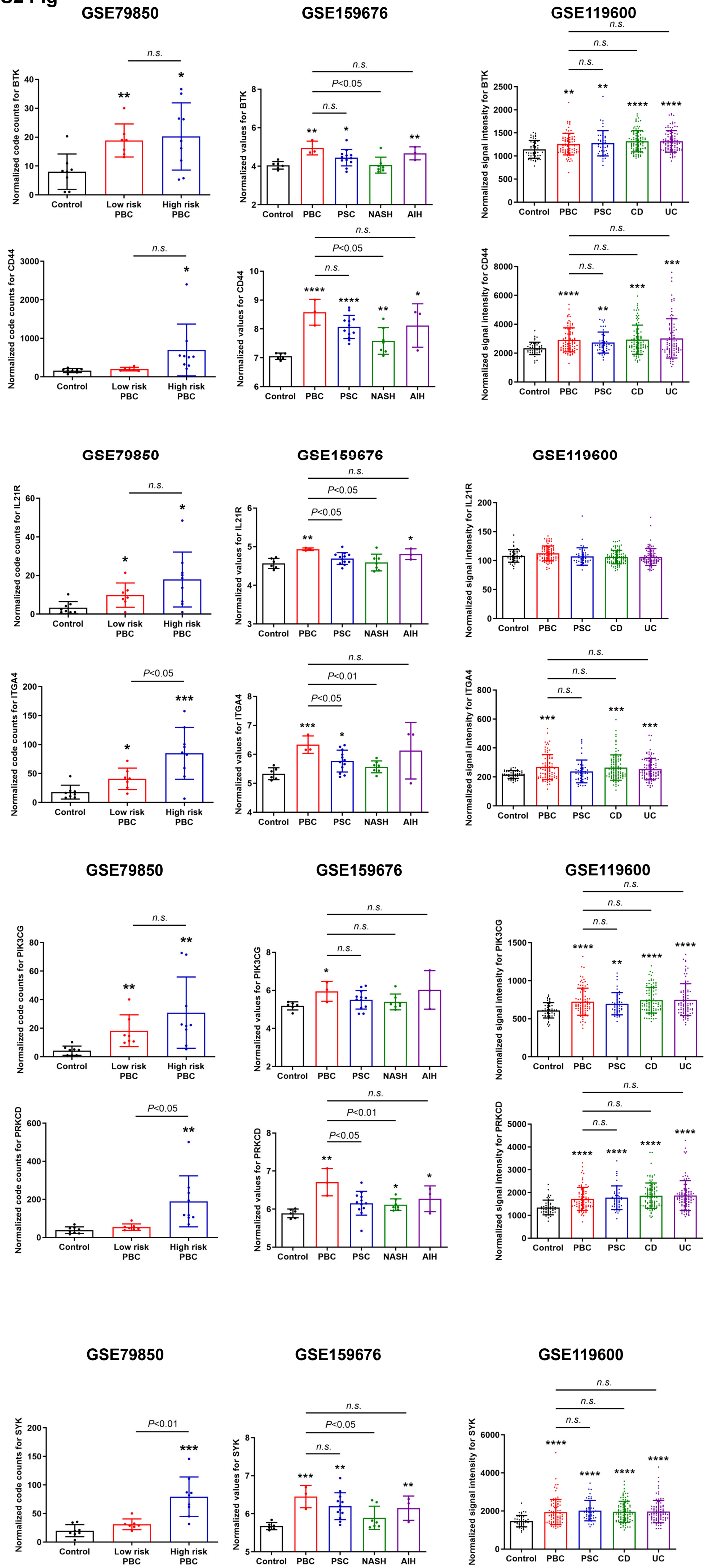

Supplement: S2 Fig — Mean±SD, *P<0.05, **P<0.01, ***P<0.001, and ****P<0.0001 compared to the Control group. (PDF) [file pone.0292998.s002.pdf]

S3 Fig

PBC vs Control

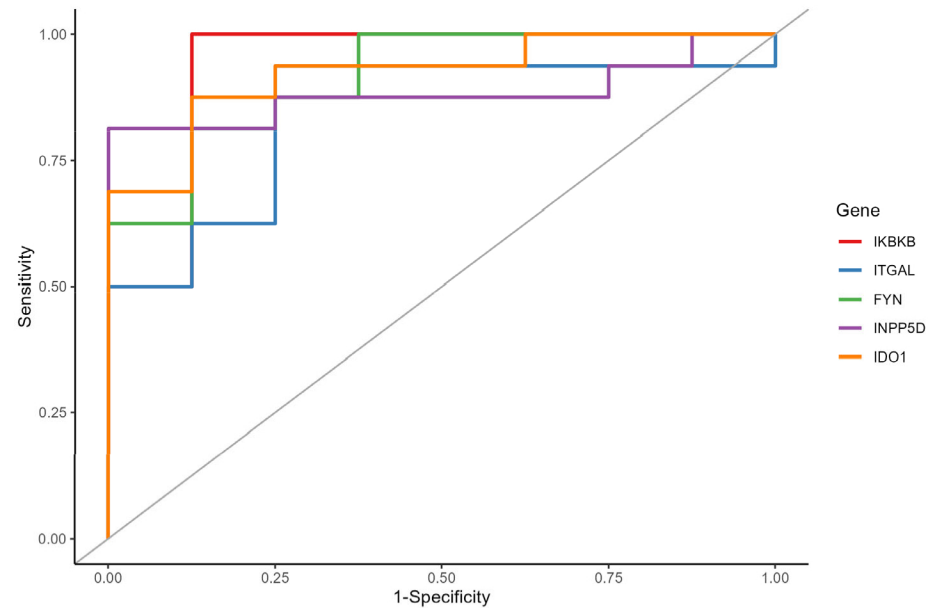

High risk PBC vs Low risk PBC

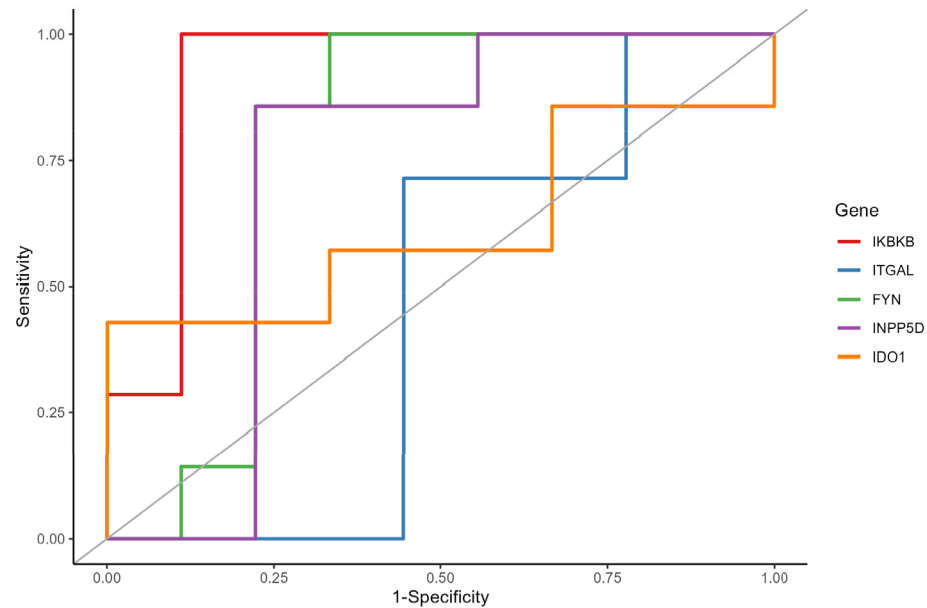

Supplement: S3 Fig — (PDF) [file pone.0292998.s003.pdf]

S4 Fig

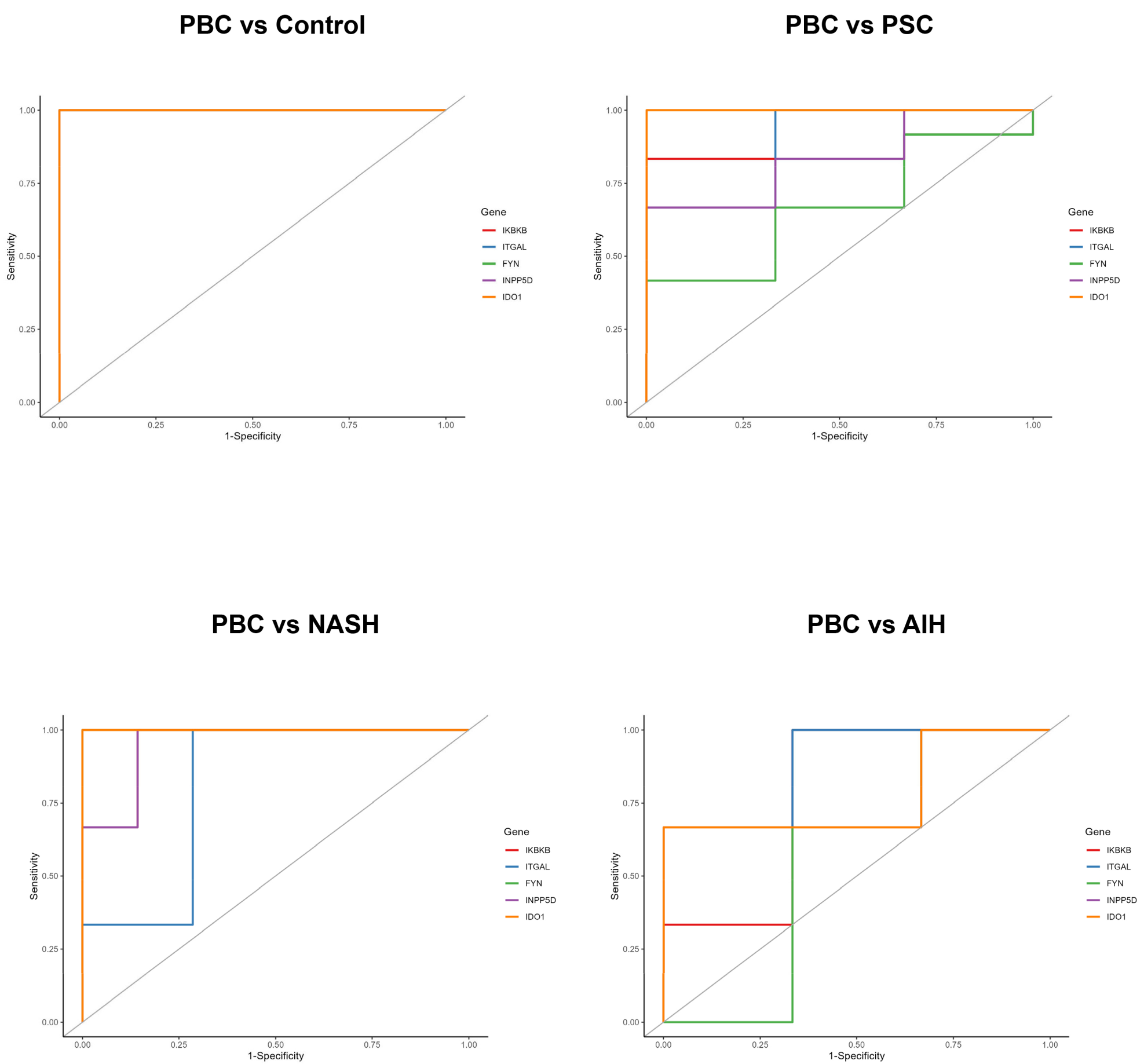

Supplement: S4 Fig — (PDF) [file pone.0292998.s004.pdf]
