# Supplementary material for: Bioinformatic analysis identified novel candidate genes with the potentials for diagnostic blood testing of primary biliary cholangitis
Source: PLoS One. 2023 Oct 16;18(10):e0292998. doi: 10.1371/journal.pone.0292998 (PMC10578581; doi:10.1371/journal.pone.0292998)
Supplement: S1 Table — (DOCX) [file pone.0292998.s005.docx]

# S1 Table. ROC curve data of GSE119600 for 12 candidate genes

|  | **PBC vs Control** | | |
| --- | --- | --- | --- |
|  | **AUC** | **Sensitivity (%)** | **Specificity (%)** |
| **BTK** | 0.6495 | 76.7 | 53.2 |
| **CD44** | 0.7161 | 60 | 74.5 |
| **FYN** | 0.6241 | 60 | 68.1 |
| **IDO1** | 0.6652 | 55.6 | 72.3 |
| **IKBKB** | 0.7721 | 77.8 | 72.3 |
| **IL21R** | 0.5979 | 55.6 | 63.8 |
| **INPP5D** | 0.7674 | 71.1 | 70.2 |
| **ITGA4** | 0.6719 | 54.4 | 87.2 |
| **ITGAL** | 0.8197 | 76.7 | 74.5 |
| **PIK3CG** | 0.7007 | 72.2 | 66.0 |
| **PRKCD** | 0.7508 | 76.7 | 70.2 |
| **SYK** | 0.74 | 64.4 | 74.5 |
|  | **PBC vs PSC** | | |
|  | **AUC** | **Sensitivity (%)** | **Specificity (%)** |
| **BTK** | 0.5057 | 48.9 | 57.8 |
| **CD44** | 0.5607 | 60 | 55.6 |
| **FYN** | 0.6264 | 64.4 | 61.1 |
| **IDO1** | 0.6146 | 68.9 | 52.2 |
| **IKBKB** | 0.5985 | 60 | 55.6 |
| **IL21R** | 0.6516 | 57.8 | 65.6 |
| **INPP5D** | 0.5956 | 48.9 | 66.7 |
| **ITGA4** | 0.6073 | 71.1 | 51.1 |
| **ITGAL** | 0.69 | 62.2 | 76.7 |
| **PIK3CG** | 0.5435 | 55.6 | 56.7 |
| **PRKCD** | 0.5351 | 55.6 | 56.7 |
| **SYK** | 0.5725 | 55.6 | 58.9 |
|  | **PBC vs CD** | | |
|  | **AUC** | **Sensitivity (%)** | **Specificity (%)** |
| **BTK** | 0.5765 | 58.9 | 55.8 |
| **CD44** | 0.5109 | 54.4 | 50.5 |
| **FYN** | 0.6701 | 62.2 | 66.3 |
| **IDO1** | 0.6516 | 68.9 | 55.8 |
| **IKBKB** | 0.5427 | 62.2 | 0.526 |
| **IL21R** | 0.6453 | 57.8 | 64.2 |
| **INPP5D** | 0.5019 | 58.9 | 48.4 |
| **ITGA4** | 0.5094 | 54.4 | 50.5 |
| **ITGAL** | 0.8102 | 78.9 | 71.6 |
| **PIK3CG** | 0.5332 | 54.4 | 53.7 |
| **PRKCD** | 0.5772 | 63.3 | 54.7 |
| **SYK** | 0.5342 | 53.3 | 57.9 |
|  | **PBC vs UC** | | |
|  | **AUC** | **Sensitivity (%)** | **Specificity (%)** |
| **BTK** | 0.5705 | 54.8 | 58.9 |
| **CD44** | 0.5302 | 53.8 | 54.4 |
| **FYN** | 0.6575 | 65.6 | 61.1 |
| **IDO1** | 0.6863 | 65.6 | 64.4 |
| **IKBKB** | 0.4858 | 57.0 | 52.2 |
| **IL21R** | 0.6595 | 57.0 | 72.2 |
| **INPP5D** | 0.5098 | 46.2 | 58.9 |
| **ITGA4** | 0.5332 | 55.9 | 54.4 |
| **ITGAL** | 0.7658 | 72.0 | 83.3 |
| **PIK3CG** | 0.519 | 51.6 | 54.4 |
| **PRKCD** | 0.5658 | 58.1 | 60 |
| **SYK** | 0.5348 | 60.2 | 51.1 |
