# Supplementary material for: Bioinformatic analysis identified novel candidate genes with the potentials for diagnostic blood testing of primary biliary cholangitis
Source: PLoS One. 2023 Oct 16;18(10):e0292998. doi: 10.1371/journal.pone.0292998 (PMC10578581; doi:10.1371/journal.pone.0292998)
Supplement: S2 Table — (DOCX) [file pone.0292998.s006.docx]

# S2 Table. ROC curve data of GSE79850 for 12 candidate genes

|  | **PBC vs Control** | | |
| --- | --- | --- | --- |
|  | **AUC** | **Sensitivity (%)** | **Specificity (%)** |
| **BTK** | 0.8438 | 87.5 | 87.5 |
| **CD44** | 0.8438 | 75.0 | 87.5 |
| **FYN** | 0.9141 | 81.2 | 87.5 |
| **IDO1** | 0.9219 | 87.5 | 87.5 |
| **IKBKB** | 0.9375 | 100 | 87.5 |
| **IL21R** | 0.8594 | 87.5 | 87.5 |
| **INPP5D** | 0.8828 | 81.2 | 100 |
| **ITGA4** | 0.8672 | 87.5 | 87.5 |
| **ITGAL** | 0.8438 | 93.8 | 75.0 |
| **PIK3CG** | 0.9688 | 93.8 | 87.5 |
| **PRKCD** | 0.8906 | 75.0 | 87.5 |
| **SYK** | 0.8828 | 93.8 | 75.0 |
|  | **High risk PBC vs Low risk PBC** | | |
|  | **AUC** | **Sensitivity (%)** | **Specificity (%)** |
| **BTK** | 0.4921 | 57.1 | 55.6 |
| **CD44** | 0.9524 | 100 | 88.9 |
| **FYN** | 0.7778 | 85.7 | 77.8 |
| **IDO1** | 0.619 | 57.1 | 66.7 |
| **IKBKB** | 0.9206 | 100 | 88.9 |
| **IL21R** | 0.6667 | 85.7 | 66.7 |
| **INPP5D** | 0.7302 | 85.7 | 77.8 |
| **ITGA4** | 0.8413 | 85.7 | 77.8 |
| **ITGAL** | 0.4603 | 71.4 | 55.6 |
| **PIK3CG** | 0.6349 | 71.4 | 77.8 |
| **PRKCD** | 0.9841 | 100 | 88.9 |
| **SYK** | 0.9524 | 85.7 | 88.9 |
