# Supplementary material for: Bioinformatic analysis identified novel candidate genes with the potentials for diagnostic blood testing of primary biliary cholangitis
Source: PLoS One. 2023 Oct 16;18(10):e0292998. doi: 10.1371/journal.pone.0292998 (PMC10578581; doi:10.1371/journal.pone.0292998)
Supplement: S3 Table — (DOCX) [file pone.0292998.s007.docx]

# S3 Table. ROC curve data of GSE159676 for 12 candidate genes

|  | **PBC vs Control** | | |
| --- | --- | --- | --- |
|  | **AUC** | **Sensitivity (%)** | **Specificity (%)** |
| **BTK** | 1 | 100 | 100 |
| **CD44** | 1 | 100 | 100 |
| **FYN** | 1 | 100 | 100 |
| **IDO1** | 1 | 100 | 100 |
| **IKBKB** | 1 | 100 | 100 |
| **IL21R** | 1 | 100 | 100 |
| **INPP5D** | 1 | 100 | 100 |
| **ITGA4** | 1 | 100 | 100 |
| **ITGAL** | 1 | 100 | 100 |
| **PIK3CG** | 0.9444 | 100 | 83.3 |
| **PRKCD** | 1 | 100 | 100 |
| **SYK** | 1 | 100 | 100 |
|  | **PBC vs PSC** | | |
|  | **AUC** | **Sensitivity (%)** | **Specificity (%)** |
| **BTK** | 0.8611 | 83.3 | 100 |
| **CD44** | 0.7778 | 83.3 | 66.7 |
| **FYN** | 0.6667 | 66.7 | 66.7 |
| **IDO1** | 1 | 100 | 100 |
| **IKBKB** | 0.8611 | 83.3 | 100 |
| **IL21R** | 0.9167 | 91.7 | 100 |
| **INPP5D** | 0.8333 | 66.7 | 100 |
| **ITGA4** | 0.8611 | 75.0 | 100 |
| **ITGAL** | 0.8889 | 66.7 | 100 |
| **PIK3CG** | 0.7222 | 83.3 | 66.7 |
| **PRKCD** | 0.8889 | 75.0 | 100 |
| **SYK** | 0.75 | 83.3 | 66.7 |
|  | **PBC vs NASH** | | |
|  | **AUC** | **Sensitivity (%)** | **Specificity (%)** |
| **BTK** | 0.9048 | 100 | 85.7 |
| **CD44** | 0.9524 | 100 | 85.7 |
| **FYN** | 1 | 100 | 100 |
| **IDO1** | 1 | 100 | 100 |
| **IKBKB** | 0.9524 | 100 | 85.7 |
| **IL21R** |  | 100 | 85.7 |
| **INPP5D** | 0.9524 | 100 | 85.7 |
| **ITGA4** | 1 | 100 | 100 |
| **ITGAL** | 0.8095 | 100 | 71.4 |
| **PIK3CG** | 0.8571 | 100 | 71.4 |
| **PRKCD** | 0.9524 | 100 | 85.7 |
| **SYK** | 0.9048 | 100 | 71.4 |
|  | **PBC vs AIH** | | |
|  | **AUC** | **Sensitivity (%)** | **Specificity (%)** |
| **BTK** | 0.7778 | 100 | 66.7 |
| **CD44** | 0.7778 | 66.7 | 100 |
| **FYN** | 0.5556 | 66.7 | 66.7 |
| **IDO1** | 0.7778 | 66.7 | 100 |
| **IKBKB** | 0.7778 | 100 | 66.7 |
| **IL21R** | 0.7778 | 100 | 66.7 |
| **INPP5D** | 0.7778 | 66.7 | 100 |
| **ITGA4** | 0.6667 | 100 | 66.7 |
| **ITGAL** | 0.8889 | 66.7 | 100 |
| **PIK3CG** | 0.5556 | 66.7 | 66.7 |
| **PRKCD** | 0.7778 | 66.7 | 100 |
| **SYK** | 0.7778 | 66.7 | 100 |
