# Supplementary material for: Bioinformatic analysis identified novel candidate genes with the potentials for diagnostic blood testing of primary biliary cholangitis
Source: PLoS One. 2023 Oct 16;18(10):e0292998. doi: 10.1371/journal.pone.0292998 (PMC10578581; doi:10.1371/journal.pone.0292998)
Supplement: Attachment — Submitted filename: Response letter.docx [file pone.0292998.s008.docx]

Our response to comments from editors and reviewers is as follows:

**Comment from the Editor:**

Please note that also Obeticholic acid and Fibrate therapy showed to have a beneficial effect on PBC.

**Our response:**

Thank you for your suggestion. We updated the introduction to introduce studies for obeticholic acid and bezafibrate (refs 5 and 6). We appreciate this comment and for providing us with the reference information.

**Comment from the Reviewer:**

In a section of 2.2 Data processing in page 12, GSE159676 contained 4829 outliers and these were excluded. In Supplementary Figure 1, box plot after excluding outliers showed that the range of gene expression is 2.0 to 3.0. However, in Figure 3, the mean value +SD for ITGAL, FYN, IKBKB, INPP5D, IDO1 are almost 6-7+1-2. These results may indicate that ITGAL, FYN, IKBKB, INPP5D, IDO1 are all outliers. Could you explain more in detail?

**Our response:**

Thank you for your comment. In short, the data values look different because the probe count values used for boxplot in Supplementary Figure 1 are log2 values; however, the values used for gene expression validation in Figure 3 and Supplementary Figure 2 are non-log2 values.

It is common to calculate and use log2 values for boxplot because it facilitates the visualization of data distribution in many cases, making it easier to interpret the results. For gene expression validation, we used non-log2 values because we wanted to show gene expression data (i.e., probe read counts) with minimal data processing. There are three data series analyzed in this study; however, each series employ different microarray platforms and data processing techniques (i.e., background subtraction, normalization, or batch correction) performed by the authors of each study. For consistency, we decided to use the original data uploaded in GEO by the authors and use them for gene expression validation in non-log2 formats. We agree with the reviewer that this could be confusing or misleading. We updated the method section and figure legends to provide clarity regarding this difference.

The rationale behind outlier removal is based on whether they are “**obviously strange**” data points. To define “obviously strange” data points to be removed and replaced by NA, we used boxplot function in R with range 10, as mentioned in the method section. For example, FYN in the GSE159676 data series has one outlier data point. The probe read count for FYN in one PSC patient is 549873 while counts for FYN in other individuals range from 6 to 8. Such a high read count of 549873 clearly indicates an anomaly or error in the data. There are many possibilities to produce these outliers, but we do not know the exact cause because the authors of the data series did not provide any clarification for this in their studies. After outlier removal and replacement, there are still many outliers found (S1 Fig), but they are not “obviously strange”, so we included these data points in our analysis. We checked data of GSE159676, and found that we only removed one outlier data from FYN and one from SYK. No outliers were found for other candidate genes including ITGAL. Outstanding outliers were identified only in GSE159676 data series, and no outliers were removed from data in GSE79850 and GSE119600.

In summary, we replaced 4,829 outliers, which accounts for less than 1% of the total data points (528,426), from GSE159676 using boxplot function in R with range 10. This is the data trimming and cleaning procedure to ensure data integrity rather than to manipulate the data or significantly alter the results. Validation of gene expression using three data series strongly supports the elevated expression of the 12 candidate genes in PBC patients. We updated the manuscript to describe this more clearly.

**Comment from the Reviewer:**

In general, needle liver biopsy is not performed in healthy controls. However, the studies in GSE79850 and GSE159676 have data of liver samples derived from healthy subjects. Will you please describe the method how to obtain liver samples in these studies, since we know that mRNA expression of liver samples markedly differs depending on the method of sample collection.

**Our response:**

Thank you for your comment. We checked the original papers and contacted the authors of the GSE79850 and GSE159676 data series. The GSE159676 authors stated that control liver tissues were obtained from patients with colorectal cancer metastasis, not healthy individuals. These liver tissues were tumor-free and used as control liver tissues, but they were not from healthy individuals. We edited the manuscript for control samples of GSE159676 to describe them as “control liver”, not healthy controls. We could not get any reply from the authors of GSE79850 including the first author and two senior authors after several contacts. In their paper, the formalin fixed paraffin embedded liver tissue samples were obtained from the cellular pathology department archive of Newcastle Hospitals NHS Foundation Trust. It seems that those samples are produced and stored in the organization in UK, and the authors probably do not know if control liver tissues were from healthy individuals or deceased donors or patients with other diseases or how these liver tissues were obtained. The authors of GSE79850 called control samples as “controls”, not “healthy controls” or “healthy individuals”. Control samples in GSE119600 are from healthy individuals because it is clearly described in the paper; however, control liver tissues of GSE79850 and GSE159676 should not be described as healthy controls. We carefully checked our descriptions and edited them correctly. We agree with the reviewer that microarray results may differ depending on the method of sample collection. We updated the section to discuss this.
