# Supplementary material for: Bioinformatic analysis identified novel candidate genes with the potentials for diagnostic blood testing of primary biliary cholangitis
Source: PLoS One. 2023 Oct 16;18(10):e0292998. doi: 10.1371/journal.pone.0292998 (PMC10578581; doi:10.1371/journal.pone.0292998)
Supplement: Attachment — Submitted filename: Response letter.docx [file pone.0292998.s009.docx]

Our response to comments from editors and reviewers is as follows:

**Comment from the Reviewer:**

The authors have replied to the first reviewer’s comments in the resubmitted version. However, one of concerns is that they have not conducted clinical study to measure ITGAL in the serum for clinical comparisons. Therefore, ITGAL gene expression was different from other patient populations but clinical utility of ITGAL has not been evidenced yet. Indeed, the authors by themselves concluded that our analysis using GEO data series identified 12 novel PBC biomarkers, among which ITGAL stands out as a particularly notable candidate, that could be useful for diagnostic blood testing. Therefore, I would recommend making careful revision of the study title and rephrasing the word of “biomarker” throughout the paper in order to prevent readers from misunderstanding and misleading.

**Our response:**

Thank you for your comment. We agree with that it could be misleading to say as if ITGAL is a novel biomarker that is useful for PBC diagnosis. We checked and proofread the entire manuscript carefully and changed description from “biomarkers” to “candidate genes” where appropriate. We also updated the abstract, discussion, and conclusion for clarity. All modifications were highlighted in the text. We appreciate your constructive feedback.
